# Analysis of the Models of Motion of Aqueous Solutions of Polymers on the Basis of Their Exact Solutions

**DOI:** 10.3390/polym10060684

**Published:** 2018-06-19

**Authors:** Oxana A. Frolovskaya, Vladislav V. Pukhnachev

**Affiliations:** Lavrentyev Institute of Hydrodynamics SB RAS, Novosibirsk State University, 630090 Novosibirsk, Russia

**Keywords:** dilute polymer solution, hereditary model, relaxation viscosity, second grade fluid

## Abstract

The qualitative properties of solutions of a hereditary model of motion of aqueous solutions of polymers, its modification in the limiting case of short relaxation times, and a similar second grade fluid model are studied. Unsteady shear flows are considered. In the first case, their properties are similar to those of motion of a usual viscous fluid. Other models can include weak discontinuities, which are retained in the course of fluid motion. Exact solutions are found by using the group analysis of the examined systems of equations. These solutions describe the fluid motion in a gap between coaxial rotating cylinders, the stagnation point flow, and the motion in a half-space induced by plane rotation (analog of the Karman vortex). The problem of motion of an aqueous solution of a polymer in a cylindrical tube under the action of a streamwise pressure gradient is considered. In this case, a flow with straight-line trajectories is possible (analog of the Hagen-Poiseuille flow). In contrast to the latter, however, the pressure in the flow considered here depends on all three spatial variables.

## 1. Introduction

It was found [1] that addition of a small number of polymers to water drastically decreases the friction drag. This effect stimulated a large cycle of experiments aimed at studying the motion of aqueous solutions of polymers in tubes and in the boundary layer in laminar and turbulent flows [2,3,4,5,6,7,8,9]. The main attention in those studies was paid to the influence of polymer additives on the flow transition from the laminar to a turbulent state with an increase in the Reynolds number. An in-depth analysis of those investigations can be found in Reference [10]. Various aspects of the dynamics of aqueous solutions of polymers were discussed in a special issue of the “Processes” journal [11]. A model of motion of polymer solutions taking into account their relaxation properties was proposed by Voitkunskii, Amfilokhiev, and Pavlovskii [12]. The sought functions in that model are the velocity vector v and the fluid pressure p. The medium is assumed to be incompressible and to have a constant density ρ and kinematic viscosity ν. The external forces are also assumed to have a potential character because they can be included into the pressure gradient in this case. Under these assumptions, the equations have the form

(1) $$\frac{dv}{dt}=-\frac{1}{\rho }\nabla p+\nu \Delta v+\frac{\kappa }{\theta }\int_{-\infty }^{t}\exp\left\{\frac{s-t}{\theta }\right\}\frac{d\Delta v}{ds}ds$$

(2) div v=0

Hereinafter, the symbol d/dt means the operator of total differentiation with respect to time, so that dv/dt=∂v/∂t+v⋅∇v. In deriving the momentum Equation (1), the authors used the following variant of the hereditary model of the Maxwell type for the stress tensor P:

(3) $$P=-pI+2\mu D+2\frac{\rho \kappa }{\theta }\int_{-\infty }^{t}\exp\left\{\frac{s-t}{\theta }\right\}\frac{d}{ds}D\;(s)\;ds$$

Here μ=ρν is the dynamic viscosity, θ is the relaxation time, κ is the normalized relaxation viscosity of dimension cm^2^ (hereafter we use the term “relaxation viscosity”), and *D* is the strain rate tensor corresponding to the vector field v. The quantities θ and μ are assumed to be constant.

If a small amount of a polymer is added to water, the solution viscosity and density remain almost unchanged, in contrast to its rheological properties. System (1), (2) contains two additional parameters: θ and κ. The relaxation time of an aqueous solution of polyacrylamide with a concentration of $10^{-2}$ percent is of the order of $10^{-4}$ s. In the case of the relaxation viscosity coefficient, the authors of the model did not provide its characteristic values, though one can assume that they are sufficiently small. At the end of the paper, we will discuss the possibility of experimental determination of this parameter.

Pavlovskii [13] replaced Equation (3) with the following equation:

(4) $$P=-pI+2\mu D+2\rho \kappa \frac{dD}{dt}$$

Equation (4) is a simplified version of Equation (3): asymptotic expansion with respect to the parameter θ→0 is performed in the integral term, and only the first term of this expansion is retained. As a result, the following momentum equation is obtained:

(5) $$\frac{dv}{dt}=-\frac{1}{\rho }\nabla p+\nu \;\Delta v+\kappa \frac{d\Delta v}{dt}$$

One more possible modification of the model of the model of motion of diluted aqueous solutions of polymers is to introduce an objective derivative of the tensor *D* [14]. In this case, the last derivative in the right-hand side of Equation (5) is replaced by the expression $2\mathrm{Div}(\tilde{d}D/dt)$, where

$$\frac{\tilde{d}D}{dt}=\frac{\partial D}{\partial t}+(v\cdot \nabla )D+DW-WD$$

W is the antisymmetric part of the tensor ∇v. As a result, we obtain a system of equations consisting of Equation (2) and

(6) $$\frac{dv}{dt}=-\frac{1}{\rho }\nabla p+\nu \Delta v+2\kappa \mathrm{Div}\left(\frac{\tilde{d}D}{dt}\right)$$

Replacement of the total derivative of the tensor *D* with respect to time by the objective derivative in Equations (2) and (5) is caused by the necessity of turning the behavior law (4) to a tensor-invariant form [14].

It is of interest to note that Equation (6) coincides with the momentum equation in the so-called second grade fluid model [15,16,17] with a special choice of parameters of this model. An analogy with the alpha-model of turbulence can be also traced [18,19]. In that model, the momentum equation has the form

(7) $$\frac{dv}{dt}=-\frac{1}{\rho }\nabla p+\nu \Delta (1-\alpha^{2}\Delta )v+\alpha^{2}\mathrm{Div}\left(\frac{\tilde{d}D}{dt}\right)$$

Here α is a small parameter with the length dimension.

The correctness of the boundary-value and initial-boundary-value problems for models of motion of aqueous solutions of polymers was analyzed in [20,21,22,23,24,25]. Much more attention was paid to the second-grade fluid (see [26] with a long list of references dealing with this topic). In particular, a number of exact solutions for this model have been studied [27,28,29,30]. As for the Pavlovskii model, a systematic study of its exact solutions began in paper [31]. The present paper contains new examples of exact solutions for both of these models. Their analysis shows that the qualitative properties of solutions described by mentioned models may differ from the solutions of the Navier-Stokes equations even at moderate Reynolds numbers.

Together with the above-mentioned models, there are alternative models in dynamics of dilute polymer solutions, particularly, the so called Oldroyd-B model; see monograph [32] and the references presented there.

## 2. Theoretical-Group Properties of Considered Models

Similar to the classical Navier-Stokes equations, the equations of model (2), (5) and two other models described in Section 1 possess many properties of symmetry. The largest Lie group admitted by system (2), (5) was calculated in Reference [31]. The basis of its infinitesimal operators is described by the formulas

$$X_{0}=\partial_{t},\;X_{kl}=x_{k}\partial_{x_{l}}-x_{l}\partial_{x_{k}}+v_{k}\partial_{v_{l}}-v_{l}\partial_{v_{k}};\;k,l=1,2,3;\;k<l,$$

$$\Phi =\varphi \;\partial_{p},\;\Psi_{k}=\psi_{k}\partial_{x_{k}}+{\dot{\psi }}_{k}\partial_{v_{k}}-\rho x_{k}{\ddot{\psi }}_{k}\partial_{p};\;k=1,2,3.$$

Here φ and $\psi_{k}$ are arbitrary functions t of the class $C^{\infty }$. The presence of arbitrary functions in the coefficients of the operators Φ and $\Psi_{k}$ is typical for many models of incompressible media (ideal and viscous fluids [33], and also the incompressible viscoelastic Maxwell model [34]). Assuming consecutively that $\Psi_{k}=1$ and $\Psi_{k}=t$, we obtain the translation operators $X_{k}=\partial_{x_{k}}$ and the Galilean translation operators $Y_{k}=t\;\partial_{x_{k}}+\partial_{v_{k}}$ along the axes $x_{k}$ (k=1,2,3). In the limiting case (κ=0), system (2), (5) transforms to the Navier-Stokes equations, and the admitted group of symmetries is extended owing to the stretch transformation with the operator

$$Z=2t\partial_{t}+\sum_{1}^{3}(x_{i}\partial_{x_{i}}-v_{i}\partial_{v_{i}})-2p\partial_{p}.$$

It is of interest that the symmetry groups admitted by all three systems where the momentum equation has the form (5), (6), or (7) coincide with each other.

Concerning the initial model (1), (2), it is rather difficult to calculate its group properties directly. Nevertheless, the integro-differential Equation (1) can be reduced to a differential equation because the kernel of the Volterra operator is degenerate. Let us differentiate Equation (1) with respect to t and eliminate the arising integral term from the resultant relation. Thus, we obtain

(8) $$\theta \frac{\partial }{\partial t}\frac{dv}{dt}+\frac{dv}{dt}=-\frac{\theta }{\rho }\frac{\partial \nabla p}{\partial t}-\frac{1}{\rho }\nabla p+\nu \theta \frac{\partial \Delta v}{\partial t}+\nu \Delta v+\kappa \frac{d\Delta v}{dt}.$$

Equation (8) does not admit the Galilean transformation, though translation over the coordinates $x_{k}$ and time, addition of an arbitrary function of time to pressure, and correlated rotations in the coordinate and velocity spaces in its group of symmetries are still possible.

The presence of a group of symmetries of the examined systems of equations allows one to construct their invariant solutions. Simple examples of such solutions are provided in the next Section. In Section 4, we construct a partially invariant (in the sense of Ovsiannikov [35]) solution of system (2), (5).

## 3. Motions with Straight-Line or Circular Trajectories

System (2), (8) is nonlinear and belongs to none of the classical types. There are no results on solvability of general initial-boundary-value problems for this system. Additional difficulties are induced by the presence of a small parameter at the highest derivative with respect to time in Equation (8) and degeneration of this equation at the solid boundary of the flow region where the no-slip condition v=0 is imposed. Nevertheless, there is a class of problems where the condition v⋅∇v≡0 is satisfied. In this case, Equation (8) transforms to a linear equation. Moreover, degeneration at the solid boundary is absent in the transformed equation.

As the first example of such problems, let us consider plane unsteady motion of an unbounded fluid with straight-line trajectories. The pressure in this flow is assumed to be constant. The velocity field has the form v=(u(y,t), 0), where the function u satisfies the equation

(9) $$\theta \frac{\partial^{2}u}{\partial t^{2}}+\frac{\partial u}{\partial t}=(\nu \theta +\kappa )\frac{\partial^{3}u}{\partial^{2}y\partial t}+\nu \frac{\partial^{2}u}{\partial y^{2}}.$$

Let us consider the Cauchy problem for this equation:

(10) $$u=u_{0}(y),\;u_{t}=u_{1}(y),\;t=0,y\in \mathbb{R}.$$

Here $u_{0}$ and $u_{1}$ are specified functions of y satisfying the natural conditions of smoothness and decreasing as y→± ∞. Problem (9), (10) reduces to the integral equation

(11) $$u(y,t)=\frac{1}{2\beta \sqrt{\pi t}}\int_{-\infty }^{\infty }u_{0}(\eta )\exp\left\{-\frac{{\left(y-\eta \right)}^{2}}{4\beta^{2}t}\right\}d\eta \;+\frac{1}{2\beta \sqrt{\pi }}\int_{0}^{t}\int_{-\infty }^{\infty }\left[\sigma u(\eta ,\tau )+\gamma \sigma e^{-\gamma \tau }\int_{0}^{\tau }e^{\gamma \varsigma }u(\eta ,\varsigma )d\varsigma +u_{1}(\eta )e^{-\gamma \tau }\right]\exp\left\{-\frac{{\left(y-\eta \right)}^{2}}{4\beta^{2}(t-\tau )}\right\}\frac{d\eta d\tau }{\sqrt{t-\tau }}$$

where the following notations are introduced: β=ν(1+ϕ), $\phi =\kappa \theta \nu^{-1}$, $\gamma ={\left[\theta (1+\phi )\right]}^{-1}$ and σ=ϕγ.

Equation (11) was derived in Reference [36], where the conditions of its unique solvability in suitable Holder weight classes were also indicated. It is convenient to use the iterative process of solving this equation for the numerical solution of the Cauchy problem (9), (10).

Assuming that θ=0 in Equation (9), we obtain the equation

(12) $$\frac{\partial u}{\partial t}=\kappa \frac{\partial^{3}u}{\partial^{2}y\partial t}+\nu \frac{\partial^{2}u}{\partial y^{2}}.$$

which describes stratified flows in the Pavlovskii model (2), (5). Only one initial condition is required here:

(13) $$u=u_{0}(y),\;t=0,\;y\in \mathbb{R}.$$

The Cauchy problem (12), (13) reduces to the integral equation

(14) $$u(y,t)=-\nu \int_{0}^{t}\int_{-\infty }^{\infty }G(y,\eta )u(\eta ,\tau )d\eta d\tau +\nu \int_{0}^{t}u(y,\tau )d\tau +u_{0}(y),$$

where

$$G(y,\;\eta )=\left\{\begin{array}{l} \frac{1}{2}\kappa^{-1/2}\exp(-\kappa^{-}{}^{1/2}y)\exp(\kappa^{-}{}^{1/2}\eta ),\;y>\eta ,\\ \frac{1}{2}\kappa^{-1/2}\exp(\kappa^{-}{}^{1/2}y)\exp(-\kappa^{-}{}^{1/2}\eta ),\;y<\eta \end{array}\right\}$$

is the Green function of the operator $L=-\kappa \frac{d^{2}}{dy^{2}}+1$ with the conditions G(y, η)→0 as y→±∞. Equation (14) is specific in the following way: it is the Volterra equation with respect to t and the Fredholm equation with respect to y.

The classical solution of problem (12), (13) satisfies the integral identity

(15) $$\int_{-\infty }^{\infty }[u^{2}(y,t)+\kappa u_{y}^{2}(y,t)]\;dy+2\nu \int_{0}^{t}\int_{-\infty }^{\infty }u_{y}^{2}(y,\tau )d\tau =\int_{-\infty }^{\infty }[u_{0}^{2}(y)+\kappa {u^{\prime }}_{0}^{2}(y)]\;dy.$$

**Figure 1:**
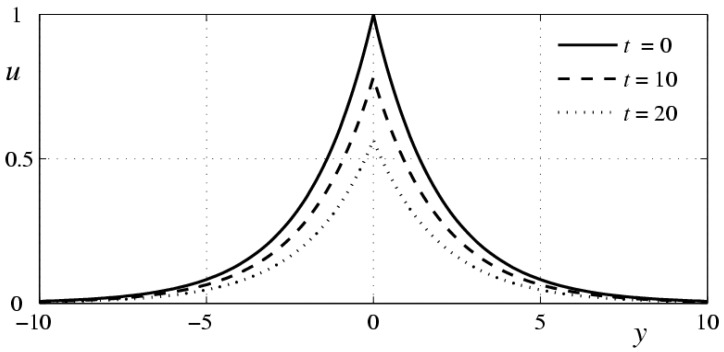
Evolution of the solution to the Cauchy problem (12), (13) with the initial function $u_{0}(y)=\exp\{-c|y|\}$ for c=0.5, $\kappa =0.25\cdot 10^{-3}$.

However, for this problem to be solved, there is no need to consider the function u as twice continuously differentiable with respect to y it is only sufficient that this function, as well as the function $u_{y}$, should be quadratically summable along the entire y axis for all t>0. This offers a possibility of proving the theorem of existence and uniqueness of the generalized solution of problem (12), (13), where the function $u_{y}$ has discontinuities at one or several points. Figure 1 illustrates the evolution of the solution to the Cauchy problem (12), (13) with the initial function $u_{0}(y)=\exp\{-c|y|\}$. Calculations were performed for the following values of the parameters: $\kappa =0.25\cdot 10^{-3}$ cm^2^, c=0.5 cm^−1^, $\nu =10^{-2}$ cm^2^/s.

Identity (15) reveals one more specific feature of Equation (12): relaxation viscosity is no longer a smoothing factor for solutions of this equation. The smoothness of the solution of the Cauchy problem with respect to the variable y remains the same as the smoothness of the initial function, whereas its smoothness along the variable t increases with time.

Let us now consider the problem of a shear flow of a second-grade fluid (2), (6). The equation for the function *u* here has the previous form (12), but the pressure starts to depend on y and t. Postulating that p→0 as y→±∞, we obtain the relation

(16) $$p=\rho \kappa u_{y}^{2}.$$

Equation (16) shows that the inclusion $u_{y}\in L^{2}(\mathbb{R})$ does not guarantee the continuity of pressure. However, if $u_{0}\in C^{k}(\mathbb{R}),\;k=1,\;2,\ldots ,$ then the solution of the Cauchy problem (12), (13) has the same smoothness along the variable y. This ensures the continuity of the function p.

**Figure 2:**
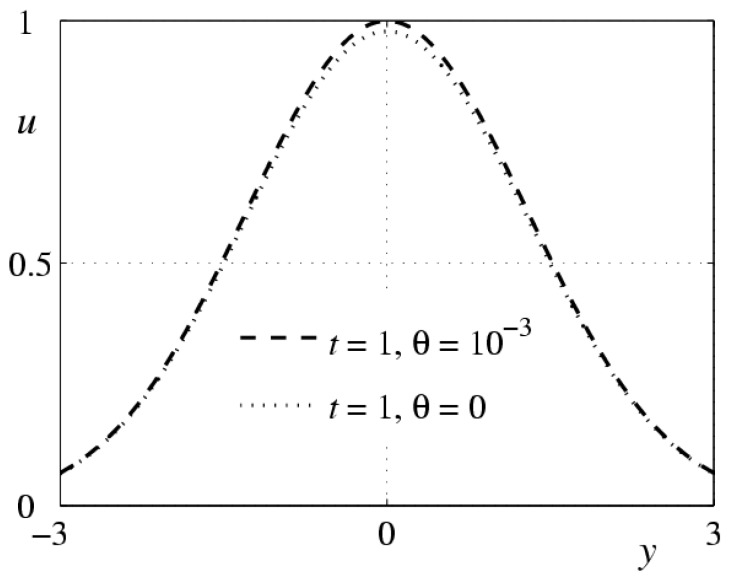
Behavior of solutions to problem (9), (10) and problem (12), (13) with the initial function $u_{0}(y)=\exp\{-dy^{2}\}$ for d=0.3.

Let us return to Equation (9) and recall that the value of θ is small. One can say that Equation (9) is a singular perturbation of Equation (12). For the solutions of the Cauchy problems (9), (10) and (12), (13) to be close to each other, it is necessary to correlate the initial function $u_{1}$ in condition (10) with the value of the derivative of $u_{t}$ of the solution of problem (12), (13) calculated at t=0. Figure 2 demonstrates the results of numerical solution of problem (9), (10) and problem (12), (13) with the initial functions $u_{0}(y)=\exp\{-dy^{2}\}$ and $u_{1}(y)=\nu (f(y)-u_{0}(y))$, where f(y) is the solution of the boundary problem

$$-\kappa f^{\prime \prime }+f=u_{0},\;f(y)\to 0\;\mathrm{as}\;y\to \pm \infty .$$

Let now the fluid fill the half-plane y>0 whose boundary is a solid surface that performs oscillations along the x axis with a velocity Vcosωt. The problem of determining the velocity u(y,t) is similar to the classical Stokes problem in viscous fluid mechanics [37]. In the model proposed by Pavlovskii, its solution has the form u=VRe[exp(iω t−ky)]. The quantities k and ω are related by the dispersion expression

(17) $$k^{2}=\frac{\omega \nu i-\kappa \omega^{2}}{\nu^{2}+\kappa^{2}\omega^{2}}.$$

We are interested in the root of Equation (17) with a positive real part,

(18) Rek=(1+γ2+γ)1/2(1+γ2)−1/2ω2ν.

where γ=κω/ν. The parameter γ is a natural similarity criterion in problems of polymer solution motion under periodic external actions. In the limit, as γ→0, Formula (18) yields the known value for the distance from the oscillating plane $l_{0}={\left(2\nu /\omega \right)}^{1/2}$ at which the amplitude of oscillations of the fluid velocity decreases by a factor of *e* [37]. The penetration depth $l_{\kappa }$ for the polymer solution is weakly affected by the relaxation viscosity if the frequency of oscillations ω is not very high. However, as γ→∞, Equation (18) predicts that Rek=κ−1/2+O(γ−1).

The solution of the Stokes problem for the hereditary model of motion of an aqueous solution of a polymer (1), (2) leads to the following dispersion relation:

$$k^{2}=\frac{\omega (1+\theta^{2}\omega^{2})\left(\nu (1+\theta^{2}\omega^{2})+\kappa \theta \omega^{2}\right)i-\kappa \omega^{2}(1+\theta^{2}\omega^{2})}{{\left(\nu (1+\theta^{2}\omega^{2})+\kappa \theta \omega^{2}\right)}^{2}+\kappa^{2}\omega^{2}}.$$

At a fixed frequency ω and a small value of the parameter θω, the roots of this equation are close to the roots of Equation (17). In what follows, we confine ourselves to considering exact solutions in the Pavlovskii model. For steady flows, this is justified by the fact that Equation (8) after multiplication by θ and limiting transition θ→0 turns to a steady variant of Equation (5). Concerning unsteady problems, the influence of the initial data on the flow evolution can be expected to attenuate with time if the process dynamics is determined by the boundary conditions of the problem.

The above-described solutions of the Cauchy and Stokes problems in models (2), (8) and (2), (5) are the simplest examples of invariant solutions of the corresponding systems of equations. One more example of the invariant solution is an analog of the classical Couette problem of polymer solution motion in a gap between coaxial rotating cylinders. The inner cylinder of radius $R_{1}$ rotates with an angular velocity Ω(t). The outer cylinder of radius $R_{2}$ does not move. The polar coordinates on the plane are denoted by r and φ, whereas $v_{r}$ and $v_{\varphi }$ are the corresponding components of the velocity vector. The motion is assumed to be planar and rotationally symmetric. In this case, only one velocity component differs from zero: $v_{\varphi }=v$. System (2), (5) reduces to one equation for the function v:

(19) $$\frac{\partial \;v}{\partial t}=\nu \left(\frac{\partial^{2}v}{\partial r^{2}}+\frac{1}{r}\frac{\partial \;v}{\partial r}-\frac{v}{r^{2}}\right)+\kappa \frac{\partial }{\partial t}\left(\frac{\partial^{2}v}{\partial r^{2}}+\frac{1}{r}\frac{\partial \;v}{\partial r}-\frac{v}{r^{2}}\right),\;R_{1}<r<R_{2},\;t>0.$$

The boundary conditions for Equation (19) follow from the no-slip condition:

(20) $$v(R_{1},t)=\Omega (t),\;v(R_{2},t)=0,\;t\geq 0.$$

Moreover, the initial condition is imposed:

(21) $$v(r,0)=v_{0}(r),\;R_{1}\leq r\leq R_{2}.$$

**Figure 3:**
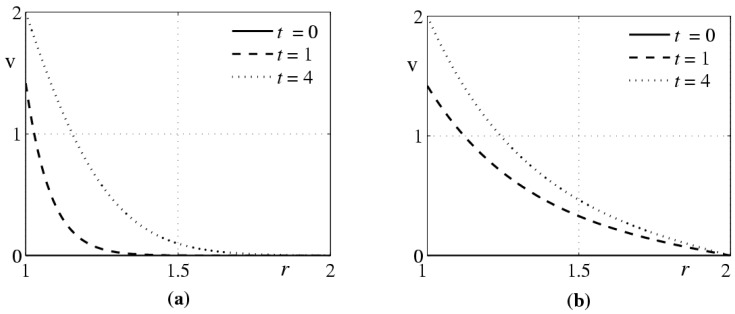
Behavior of the Couette problem solutions to (**a**) problem (19)–(21), $\kappa =0.25\cdot 10^{-2}$; (**b**) usual fluid, κ=0.

Equation (19) admits separation of variables, which allows one to construct the solution of problem (19)–(21) in the form of the Fourier series in Bessel functions and to study its qualitative properties. One of them is obvious: if the condition $\Omega (t)\to \Omega_{\infty }=const$ is satisfied as t→∞, then the flow is stabilized and transforms to the classical Couette flow [37]. The results of the numerical solution of the problem (19)–(21) are presented in Figure 3 for

$$\Omega (t)=\left\{ \begin{array}{l} 1-\cos(\omega \;t),\;t\leq \pi /\omega ,\\ 2,\;t\geq \pi /\omega , \end{array}\right.$$

and $v_{0}(r)=0$, $\nu =10^{-2}$ cm^2^/s, ω=2 s^−1^.

## 4. Motion in a Half-Space Induced by Plane Rotation

In the previous Section, we constructed invariant solutions of systems (2), (8) and (2), (5). The set of exact solutions can be extended by seeking for their partially invariant solutions in the sense of Ovsiannikov [35]. Such an example for Navier-Stokes equations is the solution of an unsteady problem of motion near the stagnation point [38]. The problem of a stagnation point flow of an aqueous solution of a polymer was studied in Reference [39] (plane steady problem), Reference [36] (plane unsteady problem), and Reference [40] (axisymmetric steady flow).

Another example of the partially invariant solution of system (2), (5) is described as

(22) $$v_{r}=rf(z),\;v_{\varphi }=rg(z),\;v_{z}=h(z),\;p=p(z),$$

where $v_{r},\;v_{\varphi }$ and $v_{z}$ are the projections of the velocity vector onto the axes of the cylindrical coordinate system r, φ, z. The functions f, g and h are found from the system

(23) $$f^{2}-g^{2}+hf^{\prime }=\nu f^{\prime \prime }+\kappa (ff^{\prime \prime }+hf^{\prime \prime \prime }),\;2fg+hg^{\prime }=\nu g^{\prime \prime }+\kappa (fg^{\prime \prime }+hg^{\prime \prime \prime }),\;2f+h^{\prime }=0,$$

The function p is found a posteriori; the equation for this function is not derived. Let us require the no-slip conditions

(24) f(0)=0,  g(0)=Ω,  h(0)=0,

to be satisfied on the plane z=0 rotating with an angular velocity Ω=const around the z axis. Moreover, the functions f and g should tend to zero as z→∞.

Solution (22) is a generalization of the classical Karman solution of Navier-Stokes equations [41,42]. The theoretical-group nature of this solution was detected in [43]. It turned out that this is a steady solution of a partially invariant submodel of Navier-Stokes equations with respect to a 5-parameter group defined by the operators $X_{1},X_{2},Y_{1},Y_{2},X_{12}$. The complete set of invariants of this group $t,\;x_{3}=z,\;v_{3}=w$, p is insufficient for constructing the invariant solution. Nevertheless, we can construct a partially invariant solution by assigning the invariant unknown functions w and p as the functions z and t. The conditions of compatibility of the resultant overdetermined system yield equations for the “extra” functions u and v. (The general procedure of obtaining partially invariant solutions was described in [35]).

As system (2), (5) also admits this group, the above-described algorithm can be applied to this system. This procedure leads to a number of new solutions for this system, and one of them yields an analog of the Karman vortex.

Let us note an important specific feature of problem (23), (24). The function h, which is a coefficient at the higher derivatives in the first two equations of system (23), has a zero point of the second order at z=0. Therefore, the question about the existence of the solution of this problem is not trivial. On the other hand, the parameter κ, which is also included into the coefficient at the higher derivatives, is a small parameter. Expansion of the problem solution into a formal power series in the parameter κ is regular and can be used as a basis of the algorithm for solving the problem numerically. This statement is supported by comparison between asymptotic behavior at infinity of the Karman vortex and solution (22). We have for the Karman vortex:

$$f=a_{0}\exp(-c_{0}\nu^{-1/2}\Omega^{1/2}z)[1+o(1)],\;g=b_{0}\exp(-c_{0}\nu^{-1/2}\Omega^{1/2}z)[1+o(1)]$$

$$h=h_{0}+O[\exp(-c_{0}\nu^{-1/2}\Omega^{1/2}z)],\;z\to \infty$$

with positive constants a, b, and $c_{0}={\left(\nu \;\Omega \right)}^{-1/2}h_{0}$. Accordingly [42], ${\left(\Omega /\nu \right)}^{1/2}h_{0}=-0.886$. Asymptotics of problem (23), (24) solutions are following:

$$f=a_{\kappa }\exp(-d_{\kappa }\nu^{-1/2}\Omega^{1/2}z)[1+o(1)],\;g=b_{\kappa }\exp(-d_{\kappa }\nu^{-1/2}\Omega^{1/2}z)[1+o(1)],$$

$$h=h_{\kappa }+O[\exp(-d_{\kappa }\nu^{-1/2}\Omega^{1/2}z)],\;z\to \infty ,$$

where

(25) $$c_{\kappa }={\left(\nu \;\Omega \right)}^{-1/2}h_{\kappa },\;d_{\kappa }=\frac{1}{2\gamma c_{\kappa }}[{\left(1+4\gamma c_{\kappa }^{2}\right)}^{1/2}-1],\;\gamma =\frac{\kappa \;\Omega }{\nu }.$$

In view of (25), $d_{\kappa }\to c_{0}$ as κ→0. The following hypothesis is likely: the Karman vortex asymptotics for dilute polymer solutions as κ→0 is uniform on the whole semi-axis z≥0. This hypothesis is confirmed by numerical solution of problem (23), (24) for small values of κ, which are not presented here. Function $d_{\kappa }(\gamma )$ decreases monotonically with growth of parameter γ and $d_{\kappa }=\gamma^{-1/2}[1+O(\gamma^{-1})]$ as γ→∞. If $c_{\kappa }=1$, which is close to $c_{0}$, and γ=3/4 then $d_{\kappa }=2/3$. This means that the relaxation viscosity influence is significant for order one values of γ: functions f and g decrease more slowly as z→∞, when the parameter κ is increasing.

The absence of the boundary layer near the solid boundary as κ→0 was previously detected in the problem of polymer solution motion near the stagnation point [36,39,40].

## 5. Motions in Cylindrical Tubes

Let us first consider the motion of an aqueous solution of a polymer with a low concentration in a cylindrical tube under the action of a given streamwise pressure gradient ∂p/∂z=c(t). If the arising flow is described by the Pavlovskii model (2), (5), it is an unsteady analog of the Poiseuille flow [37,42]. However, this is not so if model (2), (6) is used.

The examined flow is invariant with respect to the group with the operator $X=\partial_{z}+c(t)\;\partial_{p}$ admitted by system (2), (5). The general view of this solution is

(26) u=u(x,y,t), v=v(x,y,t), w=w  (x,y,t), p=c(t)z+h(x,y,t).

Here u, v and w are the projections of the velocity vector onto the x, y and z axes of the Cartesian coordinate system. The symbols Δ and ∇ are the two-dimensional Laplacian and the gradient over the variables x and y, whereas u is a two-dimensional vector with the components u and v. The subscripts in expressions such as $u_{x},\;v_{y}$ mean partial derivatives with respect to the corresponding variables.

Substitution of Equation (26) into Equations (2), (5) yields the following system of equations

(27) $$w_{t}+u\cdot \nabla w=-\rho^{-1}c(t)+\nu \Delta w+\kappa (\Delta w_{t}+u\cdot \nabla \Delta w),$$

(28) $$u_{t}+u\cdot \nabla u=-\rho^{-1}\nabla h+\nu \Delta u+\kappa (\Delta u_{t}+u\cdot \nabla \Delta u),$$

(29) ∇⋅u=0.

Let us use S to denote a bounded plane domain with a smooth boundary ∂S. The domain of the flow is the cylindrical tube Q={x, y, z: (x, y)∈S, z∈ℝ}. We have to find a solution of system (27)–(29) in the domain ω that satisfies the no-slip conditions on the domain boundary

(30) w=0,  (x,y)∈∂S, t>0,

(31) u=0,  (x,y)∈∂S, t>0,

and the initial conditions

(32) $$w=w_{0}(x,\;y),\;(x,y)\in S,\;t=0,$$

(33) $$u=u_{0}(x,\;y),\;(x,y)\in S,\;t=0.$$

If the function *c* involved into Equation (27) differs from zero, then system (27)–(29) cannot have trivial solutions w=0, u=0, h=const. However, it has solutions where u=0. In this case, the function w is a solution of the initial-boundary value problem (30), (32) for the equation

(34) $$w_{t}=-\rho^{-1}c(t)+\nu \Delta w+\kappa \Delta w_{t}.$$

Let us use $\lambda_{k}$, k=1,2,3,…, to denote the eigenvalue of the operator −Δ in the domain S with a condition of the 1st kind and $q_{k}$ to denote the corresponding eigenfunction

$$\Delta \;q_{k}+\lambda_{k}q_{k}=0,\;(x,\;y)\in S;\;q_{k}=0,\;(x,\;y)\in \partial S.$$

It is well known that the system of the functions $\left\{q_{k}\right\}$ forms an orthonormalized basis in the Sobolev space $H^{1}(S)$. The solution of problem (30), (32), (34) can be presented as the Fourier series

$$w=\sum_{1}^{\infty }w_{k}(t)\;q_{k}(x,\;y),$$

whose coefficients are determined from the ordinary differential equations

$$(1+\kappa \lambda_{k})\frac{dw_{k}}{dt}=\lambda_{k}w_{k}-\rho^{-1}e_{k}c(t),\;k=1,2,3,\ldots ;\;e_{k}=\int_{S}q_{k}dxdy$$

with the initial conditions

$$w_{k}(0)=\int_{S}w_{0}q_{k}dxdy,\;k=1,2,3,\ldots$$

Let the following conditions be satisfied: $w_{0}(x,\;y)\in H^{1}(S)$ and c(t)∈C[0, T]. Then problem (30), (32), (34) has a unique generalized solution for all T>0. Increasing the smoothness of the functions $w_{0}$, c and of the boundary of the domain S, we can prove that its generalized solution is the classical solution. These statements are proved by means of standard considerations, which are not discussed here.

Let us now assume that the function c is independent of t: c=−A=const. In this case, the problem of steady motion of a polymer solution in a tube coincides with the classical problem of hydrodynamics of a viscous fluid for determining the only nonzero velocity component w

(35) μ Δw=−A, (x, y)∈S;  w=0, (x, y)∈∂S,

where μ=ρν is the dynamic viscosity. If S is a circle of radius a, we obtain the classical Poiseuille solution

(36) $$w=\frac{A}{4\mu }(a^{2}-r^{2}),$$

where $r^{2}=x^{2}+y^{2}$.

Let us now consider the solution of system (2), (6) that is invariant with respect to the operators $\partial_{z}-A\;\partial_{p}$ and $\partial_{t}$. Its presentation has the form of Equation (26), where c=−A and the functions u, v, w and h are independent of t. The set of solutions of this system still contains solutions where u=(u,v)=0. The functions w and h are found by solving the overdetermined system of equations (35) and the equation

(37) $$h_{x}=\rho \kappa \;(2w_{x}w_{xx}+w_{y}w_{xy}+w_{x}w_{yy}),\;h_{y}=\rho \kappa \;(2w_{y}w_{yy}+w_{y}w_{xx}+w_{x}w_{xy}).$$

It turns out that system (35), (37) is compatible. A corollary of Equation (37) is the relation $w_{x}\Delta w_{y}=w_{y}\Delta w_{x}$. In turn, this relation implies that

(38) Δw=F(w)

with a certain function F. Together with Equation (35), this means that F=−A/μ=const. By virtue of Equation (35), the solution of system (37) has the form

(39) $$h=\kappa \left(\frac{\rho }{2}{\left|\nabla w\right|}^{2}-\frac{Aw}{\nu }\right)+h_{0}$$

$h_{0}=const$. In particular, by virtue of Equation (36), we obtain the following equation for a flow in a circular tube of radius a:

(40) $$h=\frac{\kappa }{8\rho }{\left(\frac{aA}{\nu }\right)}^{2}(3r^{2}-2a^{2})+h_{0}$$

Thus, in contrast to the classical Poiseuille flow, the pressure in model (2), (6) depends on all spatial variables. It should be noted that the equations of this model are identical to the equations of the second-grade fluid model under the assumptions that $\alpha_{1}+\alpha_{2}=0$ and $\alpha_{1}=\kappa$ (see [26] for more details).

If we consider the invariant solutions (26) of system (2), (6), there are still such solutions where u=0, and the function w is determined from the linear Equation (27). However, the problem of studying the overdetermined system (27), (38) does not have such a simple solution. This problem in the general form has not been solved yet, but we can give two forms of the function c(t) at which this system is compatible: $c=B_{1}\exp(k_{1}t)$ and $c=B_{2}\cos(k_{2}t)$ with constant values of $B_{1},\;B_{2},\;k_{1}$ and $\;k_{2}$.

## 6. Concluding Remarks

1. The phenomenological model of polymer solution motion formulated in Reference [12] contains two additional parameters as compared to the classical Navier-Stokes equations: relaxation time θ and relaxation viscosity κ. The first parameter can be found from experiments that are not dealing with the laminar-turbulent transition. Unfortunately, we are not aware of any reliable methods of determining the second parameter. The same problem persists if the simplified model (2), (5) is used. However, it is not a priori preferable as compared to model (2), (6), where the convective derivative of the tensor dD/dt is replaced by its objective derivative $\tilde{d}D/dt$. This dilemma could be resolved by performing an experiment where the polymer solution flows in a sufficiently long round tube under the action of a constant pressure gradient. In the first model, the classical Poiseuille flow is formed, where the pressure is independent of the radial coordinate. In the second model, Equation (40) predicts the difference in the pressures at the tube wall and at the tube axis: $\delta =3\kappa a^{2}A^{2}/8\rho \nu^{2}$. It is of interest that this effect is manifested in steady motion of the fluid.
2. Another specific feature of model (2), (6) is the absence of an unsteady analog of the Poiseuille flow with an arbitrary dependence of the pressure gradient ∂p/∂z on time. Indeed, if the function c(t) in Equation (27) has not special form, then system (27), (38) is incompatible. This means that the solution of system (2), (6) of the form (26) cannot be obtained if the velocity components u and v differ from zero, and the fluid particle trajectories are not straight lines.
3. The initial model of motion of weakly concentrated polymer solutions written in the differential form (8), (2) contains a small parameter θ at the highest derivative with respect to time. The question whether the solution of the initial-boundary-value problem for this system is close to the solution of its limiting variant as θ→0 (2), (5) is nontrivial in the general case. For stratified flows, which were considered in Section 3, it is possible to ensure the absence of the boundary layer near the plane t=0 by means of correlating the initial data for the initial and limiting problems.
4. Another problem of a singular disturbance arises in models (2), (5) and (2), (6) if the parameter κ has a small value. Here the main role belongs to the value of the similarity criterion γ=κω/ν, where ω is the frequency of the external periodic action on the fluid (or the variable of dimension 1/s, which is the angular velocity of plane rotation in the problem considered in Section 4). If the parameter γ is of the order of unity, then the qualitative differences in the behavior of the solutions for diluted polymer solutions and usual fluid may be almost invisible. However, the qualitative differences can be rather significant. This is demonstrated by the calculations of an analog of the Couette flow at the initial stage of motion
5. It was noted above that mathematical problems associated with models of aqueous solutions of polymers and second grade fluids are intensely studied. At the same time, problems of hydrodynamic stability of flows in these media have not been studied to a sufficient level. As far as we know, the problem of the Couette flow stability in such media has not been solved. It would be of interest to calculate the critical Taylor number for this problem and to analyze its behavior as κ→0.
